# New National Network of Experts (Japan Pelvic Exenteration Network: J‐PEN) Formed in a Bid to Improve Outcomes of Pelvic Exenteration in Japan

**DOI:** 10.1002/ags3.70050

**Published:** 2025-06-04

**Authors:** Hideaki Yano, Alex Mirnezami, Masataka Ikeda, Kay Uehara, Shuichiro Matoba, Yuichiro Tsukada, Toshiki Mukai, Kei Kimura, Yudai Fukui, Naoyuki Toyota

**Affiliations:** ^1^ Southampton Complex Cancer and Exenteration Team University Hospital Southampton Southampton UK; ^2^ Department of Surgery National Center for Global Health and Medicine Tokyo Japan; ^3^ Academic Surgery, Cancer Sciences University of Southampton Southampton UK; ^4^ Division of Lower GI, Department of Gastroenterological Surgery Hyogo Medical University Nishinomiya Japan; ^5^ Department of Gastroenterological Surgery Nippon Medical School Tokyo Japan; ^6^ Department of Surgery Toho University School of Medicine Tokyo Japan; ^7^ Department of Colorectal Surgery National Cancer Center Hospital East Kashiwa Japan; ^8^ Department of Gastroenterological Surgery Cancer Institute Hospital, Japanese Foundation for Cancer Research Tokyo Japan; ^9^ Department of Gastroenterological Surgery Toranomon Hospital Tokyo Japan; ^10^ Department of Colorectal Surgery Tochigi Cancer Center Utsunomiya Japan

**Keywords:** J‐PEN, locally advanced recurrent rectal cancer, locally recurrent rectal cancer, PelvEx collaborative, pelvic exenteration, UKPEN

Pelvic exenteration (PE) is a radical and extreme surgical procedure for *en bloc* removal of pelvic organs and tissues contiguously involved by cancer. PE has long been the mainstay, and often the only option to potentially provide cure, or long term control, in the management of patients with locally advanced and recurrent abdomino‐pelvic malignancies. The concept focusses on attaining an R0 cancer resection margin (most commonly defined as ≥ 1 mm), by surgically removing margin‐involved or margin‐threatened organs and structures, as this is consistently demonstrated as the most important predictor of outcome [1]. Naturally, however, such radicality comes with significant risks of complications; of loss of function and quality of life; as well as substantial healthcare resource utilisation and health economic impact. Consequently, the deployment of PE as a surgical solution has in the past been correctly cautious, but at times also overly pessimistic, informed by historically poor outcomes.

In more recent times, the PE landscape has undergone a paradigm shift. Incremental developments in multiple disciplines have helped pave the way for substantially improved outcomes in carefully selected patients. These include but are not limited to advances in diagnostic radiology; oncology; anaesthesia and peri‐operative medicine; intensive care; surgical devices and techniques; understanding of the pelvic anatomy; management and control of haemorrhage; reconstructive options; and interventional radiology [2]. As a result, the field of PE has evolved, with broadening indications and applications, and greater radicality, manifested by the fact that pelvic bones are increasingly resected as one of the most outermost tissues in a margin of concern, and reflecting the “higher and wider” approaches achievable [3].

The increasing application of PE has also emphasised some of the glaring unmet needs in the field. Examples of these are highlighted below but are not exhaustive. A lack of standardisation and differing protocols in MRI imaging techniques is one such unmet need. Poorly designed multidisciplinary team (MDT) models for the discussion of some of the most complex and heavily pre‐treated patients an MDT may receive is another such unmet need. A further concern has been in the use of surgical terminology. Contemporary PE represents an umbrella term that in the modern era encompasses a diversity of resections, and to date a confusing array of terminology has been used to describe the different surgical interventions possible. Pathological handling of specimens, for example the method of specimen orientation and marking, the number of sections taken, and management of specimens with bone, is a further area of unmet need requiring a standardisation of reporting and minimum pathological datasets. Nevertheless to date no formal international system has been described. Importantly, as a result of the lack of such radiological, surgical and pathological standardisation and definitions and quality assurance, high quality research into the field of PE has stalled, hindering innovation and further advancement of the field. A further unmet need has been the lack of a suitable forum for medical specialties that are commonly involved in PE such as urological, vascular, orthopaedic, and plastic surgery; anaesthetists; nurse specialists; and oncologists. Such a forum would promote communication, exchange of ideas and dissemination of best practice, and again serve to advance the field. Finally, a further unmet need has been a lack of opportunity to teach and train the next generation in the field.

In order to overcome these universal challenges, collaboration at national and international level is essential. To assist with this, several networks have been set up, most notably the International PelvEx Collaborative Group, and the UK‐wide PE network, UKPEN. The PelvEx Collaborative Group, which is the only global network for PE, has been holding face‐to‐face meetings annually since 2018 and contributing numerous publications to the literature [4]. UKPEN, since forming in 2019, has been implementing active initiatives to meet the aforementioned unmet needs through both online and face‐to‐face meetings. One such example of their contribution represents the now validated PE Lexicon, helping to create a common surgical language for complex pelvic cancer surgery [2, 5].

From a Japanese point of view, it is important to note that there are a number of challenges specific to Japan, beginning with terminology. Significantly, an exact equivalent in Japanese for the term “pelvic exenteration” does not exist. Kotsuban naizou zenteki (骨盤内臓全摘) is generally used but is not totally interchangeable in usage or definition with the English. There is also an absence of equivalents to key terms such as “beyond‐TME” or “extra‐anatomical resection”. Importantly, the 1 mm rule for resection margin status is also not widely accepted or endorsed by the Japanese surgical oncology community.

The treatment context for PE in Japan also differs from other countries. Historically, pelvic sidewall lymphadenectomy or dissection has been widely performed even in prophylactic settings and preoperative radiotherapy has been used more sparingly than western countries. Carbon ion radiotherapy is an increasingly applied modality to treat locally recurrent rectal cancer in Japan, and evidence for its utility is accumulating, however it is still unclear how this unique modality should be considered in the context of PE surgery. Furthermore, biological meshes, which are often used to reconstruct the defects from PE, are not currently available in Japan. Interestingly, adjunctive intraoperative treatments for advanced cancers such as intraoperative electron beam radiotherapy (IOERT) and hyperthermic intraoperative intraperitoneal chemotherapy (HIPEC) are rarely used in Japan in the context of PE, despite having been spearheaded by Japanese clinicians and researchers in the 1960s and 1970s. Taken together, these factors have made it challenging for Japanese exenterative surgeons to communicate and exchange knowledge and experience effectively with each other and international colleagues.

Against this backdrop of a globally evolving landscape and national and international unmet needs, Japanese exenterative experts gathered at PelvEx 2024 in London and, inspired by UKPEN, formed J‐PEN (Japan Pelvic Exenteration Network). The aim is to develop and formalise a network and structure in Japan that works for patients, multidisciplinary team specialists, and for all allied specialties and that can help develop national standards and quality assurance across specialties and facilitate high quality research. J‐PEN have now been holding monthly online meetings and have held case conferences in English inviting international experts at times. J‐PEN can also be instrumental in providing a platform for the next generation of Japanese surgeons to engage in discussions in English. J‐PEN's first face‐to‐face meeting is due in Tokyo in June 2025.

In conclusion, the surgical management of locally advanced and recurrent abdominopelvic cancers has undergone a paradigm shift, evolving from nihilism to cautious optimism. Radical exenterative surgery is increasingly suitable for carefully selected and very carefully counselled patients. PE is a resource intensive modality but can lead to excellent survival and good quality of life if R0 is achieved. At present several important unmet needs exist nationally and internationally, and J‐PEN hopes to tackle these through a network of centres and individuals across Japan. It takes a team to perform an exenteration, and it will take a team of units to be able to meet these unmet needs. J‐PEN is about collaboration and cooperation, not competition, to help achieve goals no individual unit or specialty can single‐handedly achieve. PE in the modern era in Japan and indeed internationally represents high‐stakes, ultra‐high risk surgery with a dearth of high quality evidence, no clear Japanese standards, guidelines, or funding envelope, and in a period of increasing management scrutiny—this status quo cannot continue.

We believe that J‐PEN can provide a unique platform for Japanese exenterative surgeons to share and enhance their understanding and experience of PE, and facilitate tackling the unmet needs in the field through national and international dialogue and collaboration.

## Author Contributions

**Hideaki Yano:** conceptualisation (equal); writing – original draft (equal); project administration (equal); writing – review and editing (equal). **Alex Mirnezami:** conceptualisation (equal); writing – original draft (equal); writing – review and editing (equal). **Masataka Ikeda:** project administration (equal); writing – review and editing (supporting). **Kay Uehara:** project administration (equal); writing – review and editing (supporting). **Shuichiro Matoba:** project administration (equal); writing – review and editing (supporting). **Yuichiro Tsukada:** project administration (equal); writing – review and editing (supporting). **Toshiki Mukai:** project administration (equal); writing – review and editing (supporting). **Kei Kimura:** project administration (equal); writing – review and editing (supporting). **Yudai Fukui:** project administration (equal); writing – review and editing (supporting). **Naoyuki Toyota:** project administration (equal); writing – review and editing (supporting).

## Ethics Statement

The authors have nothing to report.

## Consent

The authors have nothing to report.

## Conflicts of Interest

Kay Uehara is an editorial board member of Annals of Gastroenterological Surgery. The authors declare no conflicts of interest.
